# Canadian women's experiences in mixed-sex sport: Wheelchair rugby

**DOI:** 10.3389/fspor.2023.1075565

**Published:** 2023-04-21

**Authors:** Bronwyn Corrigan, Laura Misener

**Affiliations:** School of Kinesiology, Western University, London, ON, Canada

**Keywords:** wheelchair rugby, mixed-sex sport, women’s experiences, disability sport, narrative

## Abstract

Wheelchair rugby was developed in Canada in the 1970s and named an official Paralympic sport in 2000. Wheelchair rugby is one of the few Paralympic or Olympic sports that includes mixed-sex participation. Where historically women with disabilities ^1^ have had limited access to elite sporting competition, wheelchair rugby provides the opportunity for women to represent themselves as competitive and physical beings, capable of the physicality and aggressive nature of the sport alongside men. This project looks to fill the gap in research considering women's experiences in these potentially highly gendered sport settings. This study uses a thematic analysis approach to explore female athletes' lived experiences of participation in wheelchair rugby. Five provincial athletes were interviewed and shared their complex experiences competing in wheelchair rugby.

## 1. Introduction

Wheelchair rugby exploded [Fn fn1]in popularity after the documentary *Murderball* was released in the year 2005. *Murderball* documented the Canadian-American wheelchair rugby rivalry leading up to the 2004 Paralympic Games (1). Rather uniquely, wheelchair rugby is a mixed sex sport at all levels. Yet even with the popularity of the sport, little research has since been devoted to the experiences of these athletes, especially in the cases of female athletes within the sport and the ways in which gender influences their participation. This is not an anomaly, as, even more so in a mixed-sex sport there remains limited research on cis-gendered or otherwise identified women in disability sport (4–8), Thus, our work sought to expand understandings of experiences of women competing in the mixed-sex sport of wheelchair rugby. Building upon the growing literatures of women in sport experiences, this project further considers women's experiences in highly gendered sport settings contributing new insights in disability sport for females competing in a mixed-sex contact sport for persons with disabilities.

Wheelchair rugby was invented in Canada in 1977 by a group of quadriplegic athletes who wanted a sport that allowed for varying levels of upper body function (1). This makes it unique from other adapted sports, which are often created by able-bodied individuals. Traditionally female and disabled bodies are marginalized and excluded from sport; the ideal body is able and masculine (2). Individuals are taught from a young age to have a disability is to be “less than” [(9) p. 17], setting the stage for internalized ableism being instilled in childhood (9). The perspective that the non-disabled experience is dominant and preferable is the start of the definition of ableism. Ableism believes all individuals should work towards becoming as non-disabled as possible (3, 10). Indeed, non-disabled sport is considered to be worthier than disability sports in many perspectives a point that has been reflected in recent work on intersectionality of disability and gender in sport (11).

Wheelchair rugby is not integrated, meaning athletes must have a classifiable disability to compete (12). Athletes are classed from 0.5 to 3.5, wherein lower classification indicates lower levels of physical function. The classification 4.0 indicates individuals are too high functioning to compete in wheelchair rugby at international events, though they may still compete at a club level (13). Classifiers observe and test athletes' limb power and movement, trunk impairment, ball handling, and wheelchair skills to determine their classification (12). Wheelchair rugby teams are comprised of four athletes on the court at one time, where their classification is cumulatively considered. Collectively, the athletes' classifications on the court may not exceed 8.0, leading to strategic decisions about lineups (13). Men and women compete on the same teams, but in North America women receive an additional 0.5 deduction from their classification (14).

### 1.1. Literature review

Previous wheelchair rugby literature spiked in popularity after the release of the documentary *Murderball* in 2005 (1, 15–17). Gard and Fitzgerald (15) dissected disability representation in the documentary, finding that the players linked themselves to “symbols of hegemonic, straight, non-disabled masculinity” (p. 135), much like DePauw's description of the ideal body as able and masculine (2). Women are breaking barriers and taking part in masculine sports such as wheelchair rugby, but those who are successful are treated differently than men and struggle for equal support and recognition (8, 18).

Lindemann and Cherney described the hyper-masculine communication practices and culture of the sport within the documentary and analyzed how “macho” communication helped to manage disability stigma and meanings of masculinity and disability [(16), p. 108]. Females playing wheelchair rugby discussed needing to demonstrate their toughness for male teammates to accept them (16). Players described the positive aspects of the sport, particularly how more experienced teammates helped them with skills such as independent transfers (1). Playing a wheelchair-based sport allows for a sense of inclusion, and opportunities to enjoy the body for what it can do, instead of what it cannot (18). The rugby community is widely described to be one where players can ask teammates anything, especially issues they may experience that are unique to spinal cord injuries (17).

Theberge is one of the first researchers to study integration within a masculine contact sport (19). Their work focused on dismantling the binary-gender composition that determines masculinity equals athleticism (19). Gender segregation can be a method for challenging the gendered sporting body but can also act as a “vehicle for continued oppression” [(19), p. 185]. Theberge's foundational work on female ice hockey, which was severely underdeveloped compared to men's leagues, demonstrated that many talented female players look for higher levels of competition on men's or mixed-sex teams. But in doing so women competing on teams made up of both men and women reported mixed feelings: some players felt welcomed at first, then marginalized, while others had only positive experiences. Many women felt they would not be able to compete against the top men in hockey leagues for social, cultural, and physical reasons (19). There was no widespread agreement in this study to a conclusion on gender integration in sport, but underlying values held by patriarchal sport leagues do not help minorities feel welcome in a mixed-sex environment.

That work has really laid the foundation for further exploring the intersections of sport, gender and disability. Since that time, scholars have begun to explore the intersections in sport, and how these impact on the sport experience. For example, Ashton-Schaeffer and colleagues' work on wheelchair basketball demonstrated the ways in which women used ableist narrative in the sport identity while simultaneously resisting stereotypical narratives about sport and disability offering a sense of empowerment (20). In a similar vein, Richard and colleagues' study on powerchair football demonstrated how women negotiated their gender identity through norms of masculinity and ableist narratives to be accepted and feel valued in the environment (6). This is particularly interesting given the nature of powerchair sports which may not be perceived as aligning with particular masculine ideologies of aggression.

### 1.2. Theoretical framework

To frame this study, we draw upon Bourdieu's concept of practice that is comprised of three subsections: capital, habitus, and field (21, 22). Capital refers to the power an individual can hold. Social capital are individual values which raise or lower status within a specific social group (23). An athlete's gender or ability could affect social capital within a mixed-sex team based on the desired values in sport. In both non-disabled and disability sport, male athletes are considered successful the stronger, tougher, and more competitive they are (11, 18). Strong, tough men (and to a lesser extent, women) can gain capital within a sports team if those are the desirable characteristics needed to succeed. Wheelchair rugby athletes with higher classification may hold more power than athletes with lower classification. This could be reversed for women, who may hold more capital if they have lower classification due to their additional 0.5 point deduction.

The understanding that different environments lead to different internal values and norms is Bourdieu's second concept that practice is comprised of: habitus (21). Habitus is the basic lens through which people view and interact with the social world (24). The way we are raised, our social interactions, and the values we are exposed to all shape habitus. Sports inherently valuing men as holding more capital than women are a part of our habitus, something many are exposed to young and is reinforced throughout life. Organized sport has historically been a male-dominated field (25) and has worked to reinforce opposition gender norms of masculinity and femininity (26). Rugby is an ideal sport to demonstrate these masculine qualities, being an extremely physical game (16, 27, 28). Female athletes challenge gender stereotypes by participating in these types of sports but tend to be treated differently than men for doing so and struggle for equal representation and treatment (18). Qualities heralded in rugby like aggression, physicality, and power also represent forms of hegemonic masculinity, a “pattern of practice” that allows men's dominance and thus, capital, over women to continue in sport and society [(29), p. 832]. According to Ancet's work as cited in Richard and colleagues' research, men experiencing disability can be viewed as “emasculated by their supposed weakness” [(6), p. 65]. Sports, particularly those like wheelchair rugby, can be methods for regaining their sense of masculinity and capital (11, 18).

The last concept of practice is that of field (21). Field is intrinsically linked with habitus. Hierarchies of social organizations create fields, based on common interests which force groups to interact. Sport teams are a perfect example of a field. Relationships and interactions in fields are fraught with power, based on historical relations that may include patriarchal and masculine entities (24), as described previously. Historically, sports that were considered appropriate for women reflected stereotypically feminine characteristics: figure skating, synchronized swimming, and gymnastics (30). Women who are disabled face double discrimination: gender and disability. Typically, women are expected to demonstrate feminine characteristics such as gentleness and compassion (31). When considered within the context of disability, these stereotypical feminine traits tend to disappear. Cis-gendered women with disabilities are often seen as nonsexual and non-gendered (6).

Where sports like wheelchair rugby are a field for opportunities for disabled men to regain a sense of masculinity and increase their social capital, they serve no similar purpose for women with disabilities. Instead, wheelchair rugby invites athletes with differing levels of capital into the same field to complete, providing a unique experience for those who hold less capital: females, and men with lower classifications. Habitus is a concept about the unconscious systems leading to the establishment of people's internal values and norms (22), leading to the idea of ableism, which is unconsciously taught from a young age (9). Though wheelchair rugby is a mixed-sex sport and both women and men compete together on the same team, females are grossly underrepresented at the club and international stage: of the 96 wheelchair rugby athletes listed at the 2016 Paralympic Summer Games, only two were women (32). No numbers are publicly available for numbers of women competing in wheelchair rugby clubs in Canada, but anecdotal evidence with the athletes interviewed indicates there are very few.

Habitus is a critical lens to utilize in this research because sporting culture tends to reinforce gender roles learned from birth. Athletes joining a team quickly learn what role to portray in their sport field to avoid being excluded and gain capital with their teammates. The aim of this research was to better understand the female narrative of being involved in a masculine sport by framing their stories in a gendered habitus lens. This study proceeded with the understanding that gender and its associated qualities are socially constructed (27, 33), but we will use the terms female, woman/women, male, and man/men interchangeably throughout this research. This is to follow the gender-based definitions used in sporting contexts.

## 2. Methodology

An interpretive research paradigm frames this thematic analysis. Using this focus, we acknowledge that reality is socially constructed, and there are multiple meanings of the world based on individual experiences (34). The first author identifies as a non-disabled rugby player who has played senior women's full contact rugby for twelve years, thus her experience playing rugby with men was limited to touch or flag rugby. Since the time of data collection, the opportunity to play in full-contact mixed sex rugby has been important. The second author has limited experience in rugby and is not currently involved in any mixed-sex sports. Though neither author shares the same experiences as the women interviewed, their own experiences in sport have shaped the understanding of what it is like to be a female competing in a masculine sport and brought forth my own perspectives of observing hegemonic masculinity in the Canadian rugby system. We used thematic analysis to examine the lived experience of competitive female wheelchair rugby players participating in mixed-sex competition environments through analyzing transcribed interviews and manually coding for themes.

## 3. Methods

Five women who played at the provincial or national level within two years of being contacted participated in two interviews each. The small sample size is reflective of the pool of eligible athletes across Canada, as well as the intricacies of narrative work. Five athletes ensured a small enough sample to manage the rich material, yet large enough to provide new perspectives of understanding (35).

### 3.1. Participants

Emails were sent to 55 wheelchair rugby clubs, disability sport clubs, and individuals related to these organizations across Canada. All email addresses were publicly available *via* websites. The email indicated that participants were actively being recruited for this study if they had played wheelchair rugby at the provincial or national level within the past two years. Female players at competitive levels in Canada are limited, and therefore it was desirable to gain their perspective on being a minority in the sport. Of the 55 emails sent, six players responded indicating their interest in the study. Five athletes were interviewed for this study, as one athlete did not participate.

All interviews were digitally recorded and transcribed. Initial interviews were semi-structured and lasted between 26 and 65 min. Follow-up interviews were also semi-structured and based upon themes identified from the initial interviews. These lasted between 14 and 26 min.

Players reported having between three to six years of playing experience. All players currently compete at the club and provincial level. All athletes were required to be fluent in English and a minimum of thirteen years of age to participate. The demographics, as well as years played, are presented in Table 1. For privacy reasons we have not listed their age, diagnosis of disability, or whether their disability is congenital or acquired. All athletes are Canadian and have a diverse range of ethnicities. Using pseudonyms to ensure anonymity; Felicia and Gail were competitive athletes prior to sustaining spinal cord injuries; Charlotte was involved in sport but never considered herself athletic prior to playing wheelchair rugby; and Nadia and Mary were minimally involved in sport until their teen years.

**Table 1:** Summary of playing experience.

Athlete pseudonym	Years played- club	Years played- provincial	Total years played
Mary	3	2	3
Felicia	3	3	3
Charlotte	4	3	4
Nadia	6	2	6
Gail	5	2	5

### 3.2. Data collection and analysis

When potential participants emailed to indicate their interest, the author responded with an electronic copy of the Letter of Information and Consent Form and availability for times they could meet *via* Zoom or over the phone. Athletes were asked about their athletic experiences growing up, how they got involved with wheelchair rugby, their opinions on why more men than women compete in wheelchair rugby, recommendations to Wheelchair Rugby Canada to increase the number of women playing, and if they will continue to play wheelchair rugby in their future. Follow up interviews asked about their experiences with staff and coaches in the sport, other experiences they would like to share, and if they had ever want it to be a gender-based sport (i.e., separate into a women's league and a men's league). Follow up interviews also allowed for exploration of any themes or experiences that were shared in the initial interview.

Athletes were assigned pseudonyms to ensure anonymity. Certain information was not shared as it may compromise their identity due to the small population these athletes belong to. The objective of data analysis was not to find consistent themes across the athletes' diverse stories, but to analyze experiences in relation to a common factor and to identify patterns related to a common issue (35). Thematic analysis allows for threads between stories to be linked under common patterns to develop stories (36). We followed Braun, Clarke and Weate's guide for how to complete thematic analysis (36).

## 4. Results

Utilizing thematic analysis allowed insight into the layered live experiences of the five athletes. We identified commonalities across each woman's narrative and used quotes to introduce and represent each section. These sections focus on communication, the classification and points system, and the sporting culture.

### 4.1. Communication in wheelchair rugby

Athletes frequently spoke about the aggressive environment in wheelchair rugby, a quality that contributes to the culture of hegemonic masculinity. Few of the athletes interviewed started playing wheelchair rugby with any previous exposure or understanding of the sport. More experienced players, usually male, share feedback with newer players but this is often in an aggressive manner. Nadia described how she has been respected by her teammates because “I know my place, I know what I”m supposed to do, and I can be there.” She feels valued by her team because she understands her role, gaining the respect of her mostly male teammates. One of her teammates in particular frequently swore at members of the team, but called her his “super shadow” because “every time he turned around, I was there to block for him”.

Nadia continued to discuss the intensity of the sport. She acknowledged the sport is mostly made up of men and they “don’t give a shit if they hit you too hard, spin you or flip you on the ground”. According to several athletes, it is easier to play in this kind of environment if you have experienced a masculinized environment before. Athletes reported that women who joined the sport expecting to be welcomed or have their varying levels of athleticism accommodated for have quit. Nadia spoke of a woman who joined the team, but did not last long:

The girl only came out for the one year … she grew up with all females around her … so if she got hit hard or told to fucking hurry up by some of the older guys … the language they use and some of the dirty jokes they like to talk about, she's not used to [that].

However, Nadia also talked about how attractive the sport is to her because of this culture, saying that her teammates will tell her not to be lazy, and that they “don’t take your bullshit”.

A notable outlier in this theme is Mary who, like other athletes, spoke highly of her team and how welcoming they have been, particularly since her switch from an individual sport to a team-based sport. She described how “people are so nice to me … they really want to try to help me”. Mary was emphatic throughout her interviews that her teammates' honesty and vulnerability created a tight-knit group; she did not mention aggressive or masculine talk amongst the team, as other athletes spoke to. She concluded by saying that her teammates just treat each other as athletes to learn and improve from.

Felicia was involved in high-level masculine sports most of her life and was matter of fact when discussing her team culture:

Any competitive team that I've ever played on I've had to earn my spot and I've had to prove myself. If not, people don't even talk to you really because you don't have value to them, so I'm not quite sure that's a gender thing. I think definitely you're behind the 8-ball because you're a woman, you've really got to prove yourself.

Felicia grew up playing many sports where she was ignored until she had proven her worth. Though she did not think it was a gender issue, she went on to say that because she is female, she naturally had to work harder to gain her teammate's respect than male athletes would. Felicia's experience in masculine sports is clear when she talks about receiving coaching and feedback from her team: “I’m not someone that's going to sit there and get defensive and come up with excuses every time my coach tells me something, I’m going to nod my head and pull up my fucking socks”.

Charlotte mentioned she can take the criticism given to her better as she is a little older: “people yell at you and I’m not crying anymore”. She reflected on how hard on herself she is, as she thinks many women are. Charlotte acknowledged some women like to be coached differently than men, which can present a challenge in mixed-sex sport. She prefers positive encouragement, something she noted that “men don’t necessarily like … they can be a bit yelly”.

There were mixed experiences with how each athlete interacted with the masculinized communication within wheelchair rugby. Felicia, someone who had plenty of experience in masculine sports her entire life, related well to this type of communication. Mary had minimal sporting involvement in her youth and praised her teammates for their honesty and vulnerability. Nadia, though she acknowledges it takes a certain type of woman to succeed in the sport, talked about being respected while at the same time being told to “eff off” by a teammate. These women's experiences demonstrate that wheelchair rugby appears to be represented by aggressive, stereotypically masculine communication between players.

### 4.2. Classification and points

Athletes were asked about how they identity as a female within a mixed-sex sport that is dominated by men, and the complexities that accompany this role. Several shared their thoughts around the automatic 0.5 point deduction in their classification for female players, which is recognized within North America but not globally, and their thoughts about playing in a male-dominated sport.

To begin, two athletes stated that there has never been a female on the American or Canadian national team who is not a low-pointer. Felicia described that:

If you look at the history of the US or the Canadian women's team, the only players that are women that made the team are low-pointers, even like a 0.5 because then they're playing, they're a zero … the coaches call them their unicorns because we … represent zero points on the court.

Mary echoed this when talking about national team selections: “so they’re more likely to take female players in if you’re a low-pointer because that doesn’t affect the game as much”. All five women interviewed are high-level competitive athletes, having played a minimum of two years on their respective provincial teams. Two of the athletes interviewed expressed desire or interest in playing for the national team, though they acknowledged that the sport is stacked against them. The fact that only women who have been low-pointers have made the national team is one part of the uphill struggle to achieve their goals. Another challenge to not just playing on the national team but competing on a mixed-sex sport in general, is perceived as biological in nature. Felicia stated, “I’m a woman trying to gain the muscle mass of a man, which is basically impossible” and later saying that if “you put two of my arms together it's one of their arms”. This is a challenge in a sport where, according to Mary, “you need two big things: you need power and you need speed. And with how you know, female and male bodies are built, DNA-wise, genetically men just can generate more power and speed”.

Acknowledging the constant game of catch-up that female wheelchair rugby athletes play is just part of the sport. Nadia admitted “at first I didn’t like the 0.5 deduction but … it sort of levels us to their playing field … we’re still weaker, we’re slower”. Adding a further complication to this deduction is that every athlete reported having their classification changed over the years for different reasons. Targeted training to improve performance can create a negative perception of the athlete's true classification level, as Felicia is quite frustrated with:

It's okay when all the other girls get a half point off because you know, they're floating around like a pylon, and there's other girls that are having an impact on the sport it's like “whoa wait a second, there's no way that can possibly be … it's impossible they could be athletic, there's gotta be something else going on here.”

Felicia trains twice a week with her team and multiple times a week with a trainer at a gym to improve her strength and abilities on the court but described how this extra training has backfired and her classification has come into question because of it. She expressed her frustrations with the classification system, both personally and on behalf of a friend in the sport:

He [the classifier] told her that her function was a 1.5, but because she had too much dominance on the court, he had to put her up to a 2, and that was because the other girls at the time getting classed were poor athletes, whereas … she's an awesome player. So basically what's happening now is we're almost getting penalized if you're a competitive player in the sport.

Felicia also faced accusations of cheating from her own teammates:

There's not as many people I guess that have the same sort of athletic resume as I have. And I think sometimes that becomes threatening for the guys because you know, lots of the guys are going well- “if she's improving this quickly, then she has to either be cheating or she has to have more function than she [says she] does.”

Mary also discussed how her classification had changed because of one tournament where she played one of her best games of her career and that happened to be a tournament a classifier was watching her at. As a result, her classification was changed which significantly impacted her effectiveness in the sport. It took an official protest and video demonstration for Mary to be able to be re-classed to a more appropriate level. Gail spoke to being re-classed as an athlete and how this benefitted her. She joked about how “I'm a really crappy 3 but I'm a good 2.5″ (actual classifications have been changed to protect identity). Nadia said the same thing upon being re-classed: “I think I would've been played a little more harsh and gotten a little more court time if I was classed correctly in the first place”.

### 4.3. Sporting culture

Athletes spoke about how great the sport was for many reasons, but also the reality of how few women play. Some explained that this gender skewing is unique to disability sport in general as more men experience disability than women. Felicia laughed as she attempted to explain this phenomenon:

There's only like 4% representation of girls in the sport. Now obviously, there's less women in general that play male-dominated sports or contact sports, that's just in able-bodied too … the same reason there's just a smaller group of us in this sport because there's even less women that are disabled. We just think girls do less dumb shit!

Gail concurred: “I think as a statistic there are more males injured than females.” There are more men who have acquired injuries than women due to higher probability for men to engage in risky behavior, akin to Felicia's comments. Mary instead said that there are more females than originally thought, and they have quite good numbers. Despite being outnumbered, athletes were audibly excited when they spoke of opportunities to play with other females. Several athletes attended an all-female camp in Houston. Gail was one of those athletes and stated, “it was incredible … the support of it was very empowering, the environment was a very unique and positive experience”. Most athletes also expressed their hopes of being able to create a female touring team. Gail expressed her desire for a Canadian or North American touring team, while Nadia said that the one time she got to play on an all-women's line up it was “awesome”.

Athletes were asked about the mixed-sex wheelchair rugby environment, and whether they would ever see or support a future in the sport with a separate women's league. The overwhelming consensus was negative. Gail attributed this to numbers and said that “I would like to see more all-female teams, but as far as a league on our own, no, I just don't see it as a reality”. Charlotte expressed her desire to play in both mixed and women's-only wheelchair rugby, but past attempts to do this had failed due to a lack of funding. The geographical constraint of a Canadian league was also brought up as a barrier. Despite these challenges, her excitement was clear about the potential of a female-only team because “it's a different kind of woman [who plays wheelchair rugby]”.

At the same time, athletes expressed concern at affecting the spirit of wheelchair rugby if a women's league were created. Nadia was concerned at such a change, saying a female league would turn the level of the game “down a notch”. She worried everything from the speed, hits, game play, and rationale would change with a female league, and expressed concern that “a female version of this particular sport won’t attract as much media or sponsors as the male ones would”. She went on to express that if a women's team were to form, it would likely have women who have not played before and the quality of the game would decrease because the newcomers would be worried about injuries and not used to the contact. Mary acknowledged that from a biological standpoint a female league would be more fair but would affect the current dynamic of the game. Several athletes expressed that playing with men increased their skills and their level of play.

Trying wheelchair rugby for the first time can be intimidating. Both Felicia and Nadia talked about how it takes a certain type of woman to stick with the sport. Nadia grew up a self-identified tomboy which helped with fitting in. Felicia also attended the Houston all-women's camp and reported “there was a core group of like 10–15 girls who are super intense, competitive, we hit hard and we run with the guys and the rest of them just like … you know”. Felicia went on to say that she has enjoyed her experience in the sport thus far but specified that the men on the teams she has played for are welcoming and nice, but that there is a difference between being respected or having male teammates be “nice to you because you’re playing this sport and it's cute”.

Many athletes spoke to the increased independence they gained from the sport, to where they no longer needed parents to assist them at practices or games (Felicia) or were able to move to a more independent mode of wheelchair (Nadia). Felicia mentioned how it was teasing from her teammates that helped her realize her own independence:

I used to get my parents to come to my tournaments and push my [extra] chair … once they [the guys] got to know me, they started chirping me because lots of them were completely paralyzed from the waist down and they were still doing all this stuff. So it kind of motivates you to be like, “wait a second you know, I’m not totally useless, I can do lots of things”.

Nadia echoed this idea, saying that she learned so much from her teammates with regards to skills such as transferring in and out of wheelchairs, and that they are very open with sharing information. She heralded the sport for being unique in how open everyone is about sharing information and tips to become more independent and said “everyone is so open with information sharing and not because I’m a girl or because they’re boys, but more of in rugby, we’re just one of the guys”. She had experience with other adaptive sports, and said:

When I was downhill skiing and you know … you're off away from everybody else and if you fall … you only have an able-bodied person to tell you it's okay. And then you don't take that the same as from somebody who's done that, been there.

Nadia openly admitted it took time to learn skills from her teammates, saying their support and assistance helped her as she learned to navigate life “as a quad”. She continued, “I do lots of other sports now that have me in a wheelchair, but not one that I have found to be like rugby … that gives you so much information and unconditional acceptance”.

This culture of support was discussed by all the athletes. Gail stated, “I’m always humbled by the other quads with a higher level than me, or less function, who are just killing me on the court and just killing it in life”. Felicia spoke of the bond she has with her teammates, who have experienced trauma and are all “survivors”. They all ended up in a wheelchair, and “you can use it as a crutch or you can use it to make yourself stronger and to fight, and to overcome it, and still find some enjoyment in your life”.

## 5. Discussion

The athletes provided detailed insight into their experiences as competitive female wheelchair rugby players participating in a mixed-sex competition environment. The rich stories from ten interviews provided an array of varied perspectives about female athletes with disability in sport. The findings here are discussed through Bourdieu's concepts of habitus, capital, and field, and draw upon the disability sport and women's participatory research. These stories are complex and layered, and often represent a multiplicity of identities demonstrating the complexities of intersectional identities (8). Our work aims to highlight the complexity of these multi-faceted experiences for each woman a minority in their sport. Their love and passion for the game was evident throughout the results.

### 5.1. Communication in wheelchair rugby

The examples shared in the results emulates masculine traits and reflects the gendered sporting body so prevalent in sport literatures. Our work reflects that of Lindemann and Cherney's work on masculinity and communication within wheelchair rugby (16). As noted by the participants, the overwhelming characteristics of the sport are masculine, perpetuating the narrative of the gendered sporting habitus. Athletes, especially women, who play wheelchair rugby but who do not respond to aggressive forms of communication or feedback quickly leave the sport. Nadia's experience in taking critical and aggressive feedback from her teammates gained her capital because she adapted to this communication and was successful in her play, thereby leading to her continued involvement in the sport.

Sport is often used to reinforce gender norms, creating a unique position when men and women are brought together in mixed-sex sporting environments (37). Men tend to hold more capital than women in a sporting context because men's sports are viewed with more value than women's (37). This capital also plays out in the field of wheelchair rugby where masculinized communication is dominant. The athletes interviewed acknowledged that this results in a lot of swearing and gendered dirty jokes, but also honest feedback and encouragement. Though Nadia expressed frustration at times with how she was spoken to, ultimately none of the athletes problematized being yelled or sworn at, demonstrating their normalization of agressive, potentially abusive behaviours. All five women played the sport for a minimum of three years which may have acclimatized them to the hyper-masculine communication culture and demonstrates why they do not problematize a culture that allowed them to play. Women who have previously experienced or grown up in traditionally masculine environment seem to be more accustomed to this masculinized and heternormative form of communication that is widely reported to be common in wheelchair rugby (16). Two of the athletes spoke about how they had more male than female friends, and Felicia shared her experiences in masculinized sports prior to joining wheelchair rugby. These commonalities shaped their habitus, or their norms and expectations and they have experienced success in this sport in part because of their ability to engrain themselves in this culture. The athletes, except for Mary, shared the common experience of playing in a sport where teammates and coaches speak in a direct, often aggressive manner and succeeded because they confirmed this environment. They all spoke of other athletes who did not appreciate or want to be spoken to in this manner who had left the sport. The gendered and aggressive norms become commonplace for athletes as they adopt practices which support their involvement in the sport (8). It reinforces the culture and norms that women competing in mixed-sex sport experience, and that disability sport environments also reinforce gendered and ableist norms.

### 5.2. Classification and points

A key narrative from the interviews was how wheelchair rugby sets the habitus for undervaluing its female athletes. The athletes interviewed seemed to acknowledge there was a devaluing of women, particularly in classification, but accepted ‘as necessity’ it because it allowed them to play. Mary's quote about women being more likely to make the national team if they are a low-pointer because they do not affect the game as much demonstrates the lower social capital women hold in the sporting field. This links directly with Stuntz and colleagues work on mixed-sex sporting environments where in adults, men usually outperform women in physical tasks which could lead to perceived incompetence amongst as their story resonated with their shared experience of being women (38). But in this case, there are institutionalized rules that reinforce the idea of incompetence. The athletes in this study did not consider themselves incompetent compared to their male teammates, but most accept that they will never physically be able to catch up, particularly if they are classified as a high-pointer. They may never be strong enough to compete with their top male teammates who have a physical advantage. This internalized ableism reinforces the rationalization that they use when talking about their 0.5 classification deduction.

Women will, and do, outperform many men in many sport environments. A well-known example is Billie Jean King beating Bobby Riggs in their 1978 “Battle of the Sexes” tennis match (39). Felicia is an excellent example of this outperformance. However, because the sporting and in particular the wheelchair rugby habitus has dictated a belief that women cannot outperform men, Felicia deals with accusations of cheating instead of acceptance that she is an elite athlete and works to improve her skills. Men can gain capital by working on their skill and strength to increase speed and power, two qualities Mary points out are needed in wheelchair rugby. At a 0.5 or a 1.0 classification, function is low enough that there is little difference between men and women's performance. Appreciation for women's athletic ability seems to come more freely when women are lower functioning because in addition to performing similarly to other male low-pointers, they benefit the team with reducing the overall class on the court by 0.5 points, presenting the conundrum that women tend to hold more social capital with their team the lower points they represent (6). These athletes may have gained capital with their team *via* point deduction, but the sporting habitus that dictates that women cannot be as athletic as men still prevails. This is emphasized when female athletes are celebrated when they are a low-pointer because functionally they will perform similar to a male but will get an additional 0.5 taken off. Though no athlete explicitly desired a woman's wheelchair rugby league, it could be construed that classification experiences could be altered if there was an option to play in such a league, and the 0.5 deduction was removed. Gains in individual play and strength could then be rewarded differently, and experiences competing in wheelchair rugby could be radically different. However, the potential for a different experience would disrupt the sporting habitus that these women have accepted and embodied in their experiences.

### 5.3. Sporting culture

There was a contradiction in terms of desire to grow the sport of wheelchair rugby for women. Several athletes expressed the desire to field an all-women lineup at tournaments, send women's-only team on tour, and shared excitement at recently attending the all-female camp in Texas. Despite this, there was little desire for a female-only wheelchair rugby league. There seemed to be a fear amongst the women that they will lose their acceptance and sense of identity if a new female league were created. The athletes prided themselves on being able to survive in the masculinized culture of the sport, expressing concern that introducing a women's-only league would change the parts of the game they love so much. There seems to be an unspoken understanding that wheelchair rugby is a great sport, and more women should try it out, but if they do not adapt quickly to the steep learning curve then it is not for them. As Lindemann and Cherney (16) described, sport teams have strong cultures and newcomers are forced to assimilate quickly or be left out. These athletes assimilated into their team, sporting, gendered, and disability cultures to be accepted and leaving to a female-only league would thus disrupt their stable-state habitus.

Nadia and Gail explained how men tend to be stronger and faster than women, which betters their skills more than if they were in a segregated setting. This aligns with Theberge's work with female hockey players who competed on mixed-sex teams endorsed girls playing with men until they could no longer keep up, saying it would improve their skills more than playing exclusively with women (19). Theberge also discussed the conundrum that if a female-only hockey league were available, the best female players would still abandon the league to compete with men (19). There was concern from the athletes that the quality of the game would be reduced if a women's league were created, and there was also a concern that such a league would not get the same level of recognition. Habitus has informed us that women's sport is inferior to men's, demonstrated in these comments. Fink and colleagues demonstrated how sport reinforces hegemonic masculinity by using objective measures to compare men and women, unfairly favouring men (37). Within the small group of women who play, Felicia described there being only a small number who are truly competitive. She looked down on the women who could not keep up, aligning with the sporting culture she experienced on her club team.

A commonality between all interviews was the shared description of being able to fit in with the men, who make up most of the players and drive the habitus of wheelchair rugby. Though athletes voiced concerns about small grievances with their teammates, most women thoroughly enjoyed the time they spend playing with men and feel they get al.ong better with men than women. Men clearly hold more capital in the field of wheelchair rugby, as newcomers need to gain acceptance and respect from the men, which links with Lindemann and Cherney's work (16). The sport does not adapt its culture to fit different sporting personalities. It appears that women are considered to have less capital in sport and must demonstrate they have the necessary physical prowess to compete alongside the men, enough to be an asset to the team but not enough to be considered a risk to replacing a man's spot on the court. Men continue to develop bonding social capital *via* their physicality, which naturally creates barriers that excludes others, often women (1, 23). This contributes even more to women's lack of capital in wheelchair rugby and exacerbates the power divide.

There was a common message about gaining independence and learning skills from more experienced teammates throughout all the interviews. The habitus instilled in many since childhood is that the able body is “superior” [(40), p. 109]; the heterosexual, masculine body is the ideal typical (2); and habitus dictates than an impaired body has less capital than an non-disabled female. Researchers spoke of the support the wheelchair rugby community provides to its players, confirmed here through the athlete's stories (17, 18). Naturally, many athletes want to regain independence and return to being as close to the non-disabled normal as possible. They learn skills from their teammates about independent transfers, moving from a power chair to a manual chair, and overall needing less support. Athletes spoke incredulously about the days when they used to get help from parents or other loved ones. This can be encouraging to athletes who respond well to the tough-love approach shared in the results but can drive away athletes who need a different kind of encouragement. The athletes all spoke highly of their teammates helping them regain independence through feedback and support. Dependence on anyone, whether it be assistance in transferring or helping with loading and unloading extra chairs, seems to be equated to weakness and therefore results in lower capital.

Wheelchair rugby's masculinized culture is not problematized by any of the athletes because habitus dictates that this is a normalized part of the wheelchair rugby field. Players who already hold less capital because they are a minority (such as women in a mixed-sex sport) rarely speak up to change a culture. There was widespread encouragement from all athletes throughout the interviews for women to come out and try the sport, but this encouragement was situated within the male sporting habitus. This is evident in the way that Nadia insisted that this sport may not be for everyone but encouraged women to give it a try and said, “we’re just one of the guys”. This description may discourage women who want to try the sport if they planned to seek out support networks of other women, a mechanism described by Stuntz and colleagues as well as Hargreaves that females preferred (18, 38). This research offers a specific look into the experiences of women competing in mixed-sex sport, as these athletes have all encountered some level of success and stuck with the sport, despite the stories shared here.

## 6. Implications and future directions

Interviewing these five athletes gave a rich insight into their experiences, but future studies should work to interview more women. There is no specific number of athletes researchers should interview, but as per Sandelowski's recommendations, should ensure that a sample size of participants is small enough to manage the insight and data, and large enough to provide new and rich understanding of experiences (35). Additionally, future research could interview men and women who compete on the same mixed sex teams and compare their experiences and interpretation of events.

Previous literature completed on wheelchair rugby focused on the masculinity of the sport and mainly interviewed male athletes (1, 15–17). Research focused on women's participation in mixed-sex sport has fore fronted women's experiences in comparison to playing with men and was framed around perceived competence and social support in sport (38). This research offers an insight into a niche area of sport. Disability sport is often viewed as secondary to non-disabled sport, due to internalized ableism (9). Completing further research on disability sport, already considered the minority in the sport world, would be of benefit to address internalized ableism and begin to understand the experiences of these athletes. Further, interviewing a population who are considered the minority in disability sport is an important way to hear the experiences of athletes who would often go unnoticed. There are more parallels between non-disabled sport and disability sport than many realize. Female athletes in all sports can potentially relate to the need to display extraordinary talent to be accepted by male teammates, the “handicap” assigned to ensure a level playing field, and feeling pressure to fit in. Further research in this area can bridge the gap between sports and perhaps allow for more understanding and flow of resources within various sporting leagues.

The athlete narratives can serve as a tool for sport coaches, administrators, and players to reflect on the ways they may consciously or unconsciously contribute to the overwhelming masculinity of sports and the creation of the gendered sporting body. This research is an important first step to documenting and investigating the complex lived experiences of women in mixed-sex sporting environments. Future research should examine similar populations but include athletes who compete at exclusively a recreational or a club level, or those who have previously competed in the sport but have left or stopped playing.

## 7. Significance and conclusions

These findings revealed some insights into the complex lived experiences of female wheelchair rugby athletes in Canada. The lived experiences of these five athletes are by no means a representation of every female athlete in a mixed-sex sporting environment, yet their rich narratives are a start to future research in this area. The understanding that sport is gendered is central to understanding the masculine culture, or habitus, of wheelchair rugby. Traits associated with masculinity and wheelchair rugby include aggression, physicality, and power. These qualities represent forms of masculinity and the gendered sporting body, which perpetuates men's dominance over women. The athletes interviewed in this project do not problematize any of these qualities found in wheelchair rugby. They speak of the independence they have gained from the sport, the bonds between teammates, and the often-frustrating process of classification.

The athletes acknowledged inherent problems, such as the fact that no woman who is not a low-pointer has ever made the Team Canada roster. Women receive an additional 0.5 deduction off their classification and therefore low-pointer women are recruited to act as “unicorns”, a player representing zero points on the court. However, when women train to improve their game, they are often questioned about their abilities and get re-classed because they are strong athletes in addition to receiving a half point off. There is a dichotomy between wanting to improve and become better at the game but not wanting to be accused of cheating or receive a higher classification. Women are often unfairly compared to one another, having their classification questioned because another woman with their same classification is not performing as well.

Wheelchair rugby is also rife with aggressive communication practices. Athletes seemed matter of fact when talking about being yelled at to the point of tears or being told off by their male teammates. No athlete proposed any changes to this practice however, instead speaking to how they have all adjusted and become “one of the guys”, which makes putting up with this communication easier. These athletes seem to have internalized the habitus that makes aggressive communication acceptable, as demonstrated by the main social group. The women interviewed have not problematized this practice because of desire to gain capital and fit into the habitus of the team.

It is notable that athletes from different clubs and provinces report similar communicative practices, signaling the strong gendered sporting body that appears to have dominated the sport since its inception in 1977. Bourdieu's concepts of habitus, capital and field can be clearly seen here. Habitus- values and norms individuals grow up with- dominated the discussion in this study. When individuals with similar values and interests come together, such as in the development of a sport, a strong sporting habitus is developed. The sport had a strong habitus from its inception as a physically demanding sport filled with contact and aggression. Though it is a mixed-sex sport, wheelchair rugby appears to have done little to adjust its habitus to welcome athletes who may not identify with the values of the original creators. Sports traditionally have extremely strong cultures that newcomers are forced to adopt or be left out and treated like an outsider (16).

Overall, this research presented the complex lived experiences of female wheelchair rugby athletes competing in a mixed-sex sporting competition. There is no one answer or story that is representative of every woman's experience playing wheelchair rugby, though there are certainly similarities. The research and stories shared provide a glimpse into the complexities of these athletes' experiences. Though wheelchair rugby is mixed-sex, men and women are not equally represented and women are subject to the dominant masculinity of the sport, having to either join and assimilate into the culture, be considered an outsider by their teammates, or quit.

## Data Availability

The original contributions presented in the study are included in the article/Supplementary Material, further inquiries can be directed to the corresponding author/s.

## Footnotes

^1^
Recognizing the contested language of disability, we employ person first language in this paper as it is predominant in Canada, the country of study.
